# Kinetics of Microcystin-LR Removal in a Real Lake Water by UV/H_2_O_2_ Treatment and Analysis of Specific Energy Consumption

**DOI:** 10.3390/toxins12120810

**Published:** 2020-12-21

**Authors:** Sabrina Sorlini, Carlo Collivignarelli, Marco Carnevale Miino, Francesca Maria Caccamo, Maria Cristina Collivignarelli

**Affiliations:** 1 Department of Civil, Environmental, Architectural Engineering and Mathematics, University of Brescia, 25123 Brescia, Italy; carlo.collivignarelli@unibs.it; 2 Department of Civil Engineering and Architecture, University of Pavia, 27100 Pavia, Italy; marco.carnevalemiino01@universitadipavia.it (M.C.M.); francescamaria.caccamo01@universitadipavia.it (F.M.C.); mcristina.collivignarelli@unipv.it (M.C.C.); 3 Interdepartmental Centre for Water Research, University of Pavia, 27100 Pavia, Italy

**Keywords:** cyanobacteria, cyanotoxins, drinking water, AOPs, hydrogen peroxide, algal bloom, microcystin-LR

## Abstract

The hepatotoxin microcystin-LR (MC-LR) represents one of the most toxic cyanotoxins for human health. Considering its harmful effect, the World Health Organization recommended a limit in drinking water (DW) of 1 µg L^−1^. Due to the ineffectiveness of conventional treatments present in DW treatment plants against MC-LR, advanced oxidation processes (AOPs) are gaining interest due to the high redox potential of the OH^•^ radicals. In this work UV/H_2_O_2_ was applied to a real lake water to remove MC-LR. The kinetics of the UV/H_2_O_2_ were compared with those of UV and H_2_O_2_ showing the following result: UV/H_2_O_2_ > UV > H_2_O_2_. Within the range of H_2_O_2_ tested (0–0.9 mM), the results showed that H_2_O_2_ concentration and the removal kinetics followed an increasing quadratic relation. By increasing the initial concentration of H_2_O_2_, the consumption of oxidant also increased but, in terms of MC-LR degraded for H_2_O_2_ dosed, the removal efficiency decreased. As the initial MC-LR initial concentration increased, the removal kinetics increased up to a limit concentration (80 µg L^−1^) in which the presence of high amounts of the toxin slowed down the process. Operating with UV fluence lower than 950 mJ cm^−2^, UV alone minimized the specific energy consumption required. UV/H_2_O_2_ (0.3 mM) and UV/H_2_O_2_ (0.9 mM) were the most advantageous combination when operating with UV fluence of 950–1400 mJ cm^−2^ and higher than 1400 mJ cm^−2^, respectively.

## 1. Introduction

Microcystin-LR (MC-LR) is a hepatotoxin produced by cyanobacteria such as *Microcystis aeruginosa*, *Planktothrix*, *Nostoc* and *Anabaea* and represents one of the most common and most toxic cyanotoxins for human health [1,2,3]. Cyanobacteria growth is enhanced in the presence of particular conditions such as mild temperature of water (25–35 °C), low flow rates, high concentration of nitrogen and phosphorous [4,5]. Therefore, lakes in areas with a temperate and warm climate represent a perfect habitat for their growth.

Cyanotoxins can interact and alter different parts of human metabolism with consequent effects on health of varying severity. For example, all cyanobacteria genera can produced cyanotoxins belonging to the group of Lipopolysaccharides, which have only a potential irritating effect on the tissues they have come into contact with [6]. On the other hand, the microcystins and nodularins, belonging to the group of cyclic peptides, have the liver as their main target of action being able to cross cell membranes mainly through the bile acid transporter [2,6]. Several studies highlighted the effects on liver tissue in humans exposed chronically to MC-LR [7,8,9].

The effect of microcystins was also studied by Zhou et al. [10]. They identified that the incidence rate of colorectal cancer was significantly higher in the population who drank water with high concentration of microcystins (e.g., river water) than those who drank tap water [10]. This harmful effect on intestinal cells was also confirmed by subsequent studies [11]. Alosman et al. [12] also pointed out that, aside the liver, MC-LR can cause also cardiogenic complications even if standardized animal models would be needed before the cardiotoxicity of the toxin can be defined with certainty.

The ingestion/inhalation of contaminated water in recreation (e.g., watersports) and, above all, the consumption of contaminated drinking water (DW) represent the main routes of exposure of humans to the toxin [2,6]. The effects due to secondary exposure, such as those due to the presence of MC-LR in plants and vegetables irrigated with water rich in toxins, are also being studied and quantified [13,14].

Considering the harmful effect of MC-LR revealed in literature results, the International Agency for Research on Cancer (IARC) classified this cyanotoxin as possible carcinogenic to humans (Group 2B) [6]. Based on this classification, the World Health Organization (WHO) included the MC-LR within the parameters to be monitored in DW, recommending a temporary limit of 1 µg L^−1^ for total MC-LR (free plus cell-bound) [2]. The European Union implemented this recommendation by including the MC-LR in the revision of the Drinking Water Directive in 2018, providing for a limit of 1 µg L^−1^ [15]. At the current date (15 December 2020) the proposed revision of the directive has not yet been approved so there is currently no unitary legislation at the European Union level about the presence of this toxin in DW.

However, several EU countries where the presence of cyanotoxins within surface water bodies is more widespread have already legislated on the matter providing national limits. For example, in 2012, Italy introduced a limit of 1 µg L^−1^ as equivalent MC-LR referring to the sum of the concentrations of the different microcystins congeners present in DW [16]. In France, in 2001 a decree set the limit of 1 µg L^−1^ for MC-LR in DW [17]. Instead, in 2007, this limit was referred to the sum of the microcystins quantified in the sample [17,18].

Even some non-European countries promoted laws or guidelines in order to minimize the risks for human health related to cyanobacteria and cyanotoxins in DW. For example, Canada established legislative limits for DW, with a seasonal maximum acceptable concentration of 1.5 µg L^−1^ for total microcystins [19], and provided a draft of guidelines for recreational water quality a maximum acceptable concentration of 10 µg L^−1^ for total microcystins [20]. Australia has provided non-mandatory guidelines suggesting that the concentration of total microcystins in DW should not exceed 1.3 µg L^−1^ expressed as microcystin-LR toxicity equivalents [21].

There are two possible approaches to address the problem of the presence of MC-LR in waters: (i) remove the cyanobacteria that produce them or (ii) directly eliminate the free toxin [1]. The conventional treatments present in a drinking water treatment plant (DWTP; e.g., coagulation, flocculation, sedimentation and filtration) allow one to implement the first of the two approaches [22,23,24,25]. However, these treatments are not able to remove the MC-LR already secreted by cyanobacteria and present in the water in dissolved form [26]. On the contrary, adsorption on AC is confirmed to be a viable solution for the removal of low molecular weight substances such as cyanotoxins in general and specifically MC-LR [27,28,29].

Recently, even advanced oxidation processes (AOPs) are gaining interest in the removal of MC-LR due to the high redox potential of the hydroxyl radicals (OH^•^) or sulfate radicals (SO_4_^•−^) developed in the process, which allows one to overcome some limitations given by the limited oxidizing power of other oxidizing agents towards MC-LR, such as H_2_O_2_ [30,31].

UV/H_2_O_2_ represents one of the AOPs. The main advantages are given by the absence of chemical sludge production (as opposed for instance to the Fenton process [32]), by the absence of toxic DBPs formation (unlike processes that involve chlorine and ozone [33,34,35,36]), and by the great ease of finding the oxidants used (as opposed to processes involving the use of nanostructured metals [37]).

In literature, several examples of application of this process for the removal of MC-LR are reported. UV lamps are used that emit at 254 nm of wavelength [38], close to the wavelength of maximum absorption of the MC-LR (235–238 nm [38,39]) or at 268 nm of wavelength [40]. However, most of the experiments involved the use of synthetic waters, thus only partially evaluating the combined effect of the presence of scavenger substances in the process such as the carbonates. On the contrary, this paper aims to evaluate the effectiveness of UV/H_2_O_2_ on a real lake water studying kinetics of free MC-LR removal to understand the influence of UV fluence, H_2_O_2_ dosage and initial MC-LR concentration on the process effectiveness. Moreover, the total specific energy consumption of UV/H_2_O_2_ for MC-LR removal has been evaluated and compared with UV alone to find the optimal operational conditions.

## 2. Results and Discussion

### 2.1. Effect of the Oxidant

**Figure 1:**
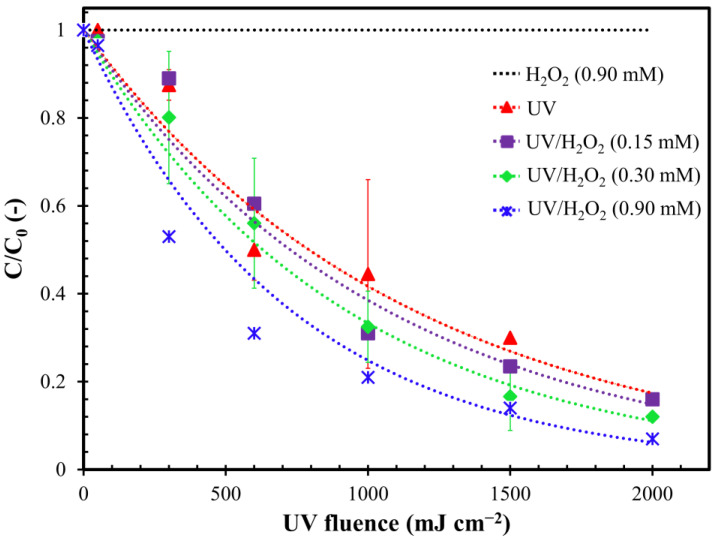
Degradation of MC-LR as a function of UV fluence in H_2_O_2_ alone, UV and UV/H_2_O_2_ processes. The colored curves represent exponential decay curve fitting. Error bars represent the confidence interval (*n* = 3). In case of the H_2_O_2_ alone treatment, the samples were taken at the time interval corresponding to the same UV fluence of the other tests. Conditions: MC-LR_0_ = 50 µg L^−1^; pH = 7.5 and fluence rate = 0.2 mW cm^−2^.

The effectiveness of the H_2_O_2_, UV and UV/H_2_O_2_ processes for MC-LR removal was investigated. Figure 1 shows the degradation of MC-LR as a function of the UV fluence using different oxidants.

H_2_O_2_ alone did not allow us to remove the toxin even with high contact time. This result is confirmed by Liu et al. [40] who observed an almost absent removal of dissolved MC-LR (0.1 µM) using H_2_O_2_ (0.1 mM) at pH nearly 7. On the contrary, the photolysis treatment with UV-C was found to be weakly effective in removing MC-LR (about 50%), with UV fluence equal to or lower than 1000 mJ cm^−2^. The toxin removal enhanced to 80% using the maximum UV fluence tested (2000 mJ cm^−2^).

The UV/H_2_O_2_ combination ensured removal yields higher than 90% with UV fluence equal to 2000 mJ cm^−2^ and H_2_O_2_ concentration of 0.9 mM. This result can be attributed to the production of OH^•^ radicals capable of almost completely oxidizing the MC-LR due to their high redox potential [41]. He et al. [38] confirmed the higher effectiveness of UV/H_2_O_2_ with respect with UV alone, obtaining more than 90% of MC-LR removal after 80 mJ cm^−2^ of fluence dose (H_2_O_2_: 1.76 mM) compared to around 20% obtained with UV alone. On the contrary to He et al. study [38], in the present work, a lower MC-LR removal was obtained using the same fluence dose, probably due to the higher initial pH that may have favored the scavenging effect on OH^•^ production [42].

### 2.2. Influence of H_2_O_2_ Dosage

**Figure 2:**
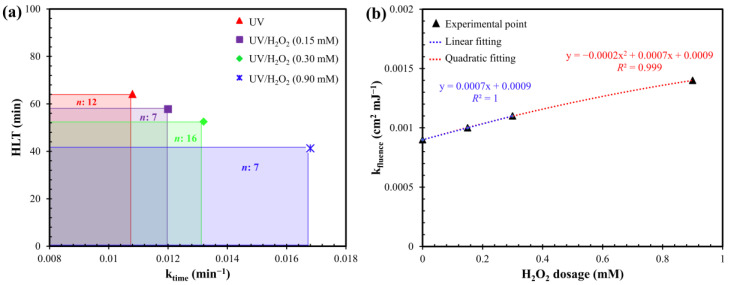
(**a**) First-order kinetic constant (k_time_) and half-life time (HLT) during degradation by UV and UV/H_2_O_2_. (**b**) First-order kinetic constant (k_fluence_) as a function of the H_2_O_2_ dosage. Conditions: MC-LR_0_ = 50 µg L^−1^; pH = 7.5; fluence rate = 0.2 mW cm^−2^ and *n*: number of data.

**Figure A1:**
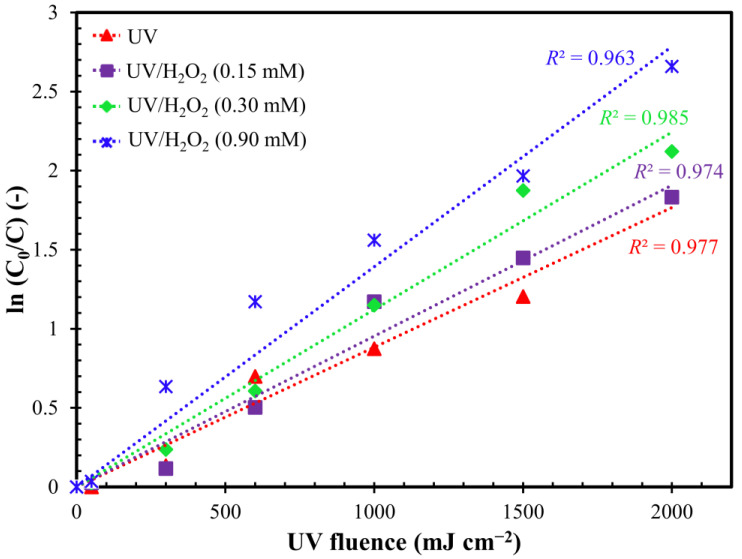
Normalized MC-LR concentration decay following a first-order kinetics model in UV alone and UV/H_2_O_2_ processes. The colored lines represented curve fitting. Conditions: [MC-LR]_0_ = 50 µg L^−1^; pH = 7.5 and fluence rate = 0.2 mW cm^−2^.

The influence H_2_O_2_ dosage on the kinetics of MC-LR removal was studied. As shown in Figure A1 in the Appendix A, all results were well fitted by applying the first-order kinetics model to calculate the MC-LR removal kinetic constants (Figure 2a and Table A1 in Appendix A). As already shown in Figure 1, the H_2_O_2_ dosage generally allowed a better removal yield of the MC-LR. However, this result appeared to be dependent on the concentration of H_2_O_2_ dosed in the reaction. In fact, the UV/H_2_O_2_ combination ensured an efficacy in removing the toxin directly proportional to the quantity of chemical oxidant dosed. The half-life time (HLT) of the MC-LR was reduced from 64.2 (UV alone) to 57.8 min (−10%), 52.5 min (−18%) and 41.3 min (−36%) in the case of UV/H_2_O_2_ (0.15 mM), UV/H_2_O_2_ (0.30 mM) and UV/H_2_O_2_ (0.90 mM), respectively.

This relation was even more evident by comparing the apparent constant rate of the process (expressed as k_fluence_) as a function of the H_2_O_2_ dosage. In Figure 2b it can be observed that, for low dosages of H_2_O_2_ (≤0.3 mM), the increase of removal kinetics can be perfectly linearly fitted (*R*^2^ = 1). The increase in the kinetics of MC-LR removal was attributable to the increase in OH^•^ production due to the initial higher concentration of H_2_O_2_ as already seen for anatoxin-a removal [43]. This result is in agreement with He et al. [38] who studied MC-LR removal from synthetic DW using UV/H_2_O_2_. They highlighted that, with initial H_2_O_2_ concentrations below 1 mM, the MC-LR degradation rate constant seemed to increase proportionally with the chemical oxidant concentration following a linear relation [38].

However, considering also a higher H_2_O_2_ dosages (0.9 mM), the best fitting has been obtained with a quadratic function (*R^2^* > 0.99). In fact, when the H_2_O_2_ concentration reached high level (1 mM for [38] or more than 3 mM for [42]), the production of OH^•^ could be inhibited due to scavenging effect and the removal of MC-LR could remain almost constant or decrease [38,42].

Compared to results obtained by He et al. [38] and Loaiza-González [44], in the present work, lower removal kinetics were obtained using the same concentration of oxidizing agent. This could be related with the presence in the real lake water of higher concentrations of carbonates (Table A3 in Appendix A), which have a high scavenger effect, unlike chlorides and sulphates [38].

**Figure 3:**
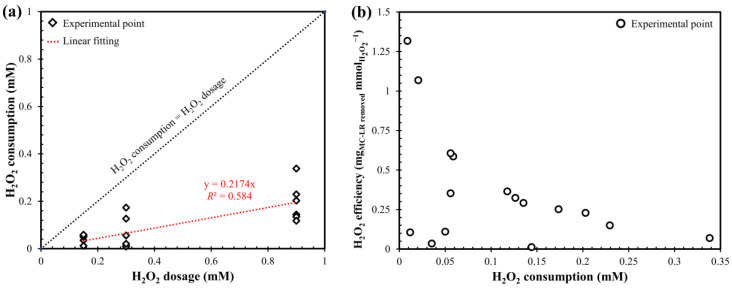
(**a**) H_2_O_2_ consumption as a function of H_2_O_2_ dosage. (**b**) H_2_O_2_ efficiency as a function of H_2_O_2_ consumption. Conditions: MC-LR_0_ = 50 µg L^−1^; pH = 7.5 and fluence rate = 0.2 mW cm^−2^.

Furthermore, the amount of oxidant consumed in the UV/H_2_O_2_ process and the H_2_O_2_ efficiency in removing MC-LR were evaluated. By increasing the initial concentration of H_2_O_2_, the consumption of oxidant also increased (Figure 3a). This can explain the kinetics detailed in Figure 2b: for constant UV fluence, higher H_2_O_2_ consumption means higher OH^•^ production and therefore higher MC-LR removal. However, as the H_2_O_2_ consumed increased, the removal efficiency of the MC-LR decreased, in terms of MC-LR degraded for H_2_O_2_ dosed (Figure 3b). This result was also observed by Penru et al. [45] in the application of the UV/H_2_O_2_ for the removal of organic matter and it was attributed to the scavenging effect of hydroxyl radicals that can limit the oxidative power of the process.

### 2.3. Influence of Initial MC-LR Concentration

**Figure 4:**
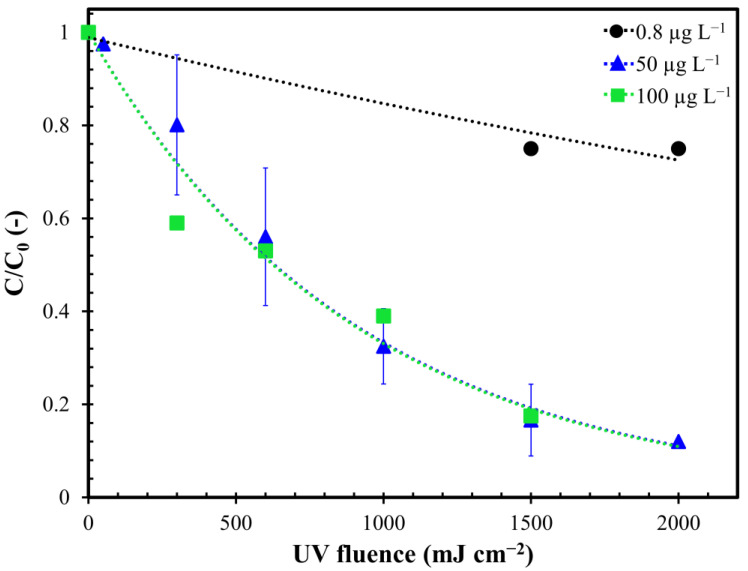
Degradation of MC-LR as a function of UV fluence in UV/H_2_O_2_ processes with different initial toxin concentration. The colored curves represent exponential decay curve fitting. Error bars represent the confidence interval (*n* = 3). Conditions: H_2_O_2_ dosage = 0.3 mM; pH = 7.5 and fluence rate = 0.2 mW cm^−2^.

Investigations on UV/H_2_O_2_ effectiveness were repeated keeping the H_2_O_2_ dosage constant and varying the initial concentration of MC-LR. The tests were conducted on real water with the addition of MC-LR to obtain a concentration of 0.8 µg L^−1^, 50 µg L^−1^ and 100 µg L^−1^. Increasing the initial MC-LR concentration from 0.8 to 50 µg L^−1^, the removal yields enhanced from 25% to 87.5% (Figure 4). Further increasing the initial MC-LR concentration to 100 µg L^−1^ did not result in an enhancement in toxin removal yields.

**Figure A2:**
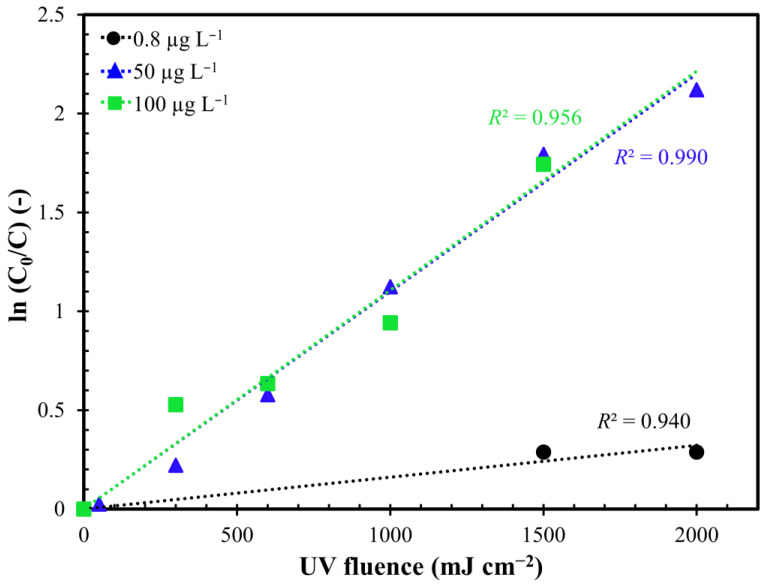
Normalized MC-LR concentration decay following a first-order kinetics model in UV/H_2_O_2_ processes with different initial MC-LR concentration. The colored lines represented curve fitting. Conditions: H_2_O_2_ dosage = 0.3 mM; pH = 7.5 and fluence rate = 0.2 mW cm^−2^.

As shown in Figure A2 in Appendix A, all results were well fitted by applying the first-order kinetics model to calculate the MC-LR removal kinetic constants (Table A2 in Appendix A). By increasing the initial concentration of toxin from 0.8 to 50 µg L^−1^, HLT decreased by about 82% (from 288.8 to 52.5 min). The further increase in the initial MC-LR concentration to 100 µg L^−1^ did not lead to a change in the removal kinetics.

**Figure 5:**
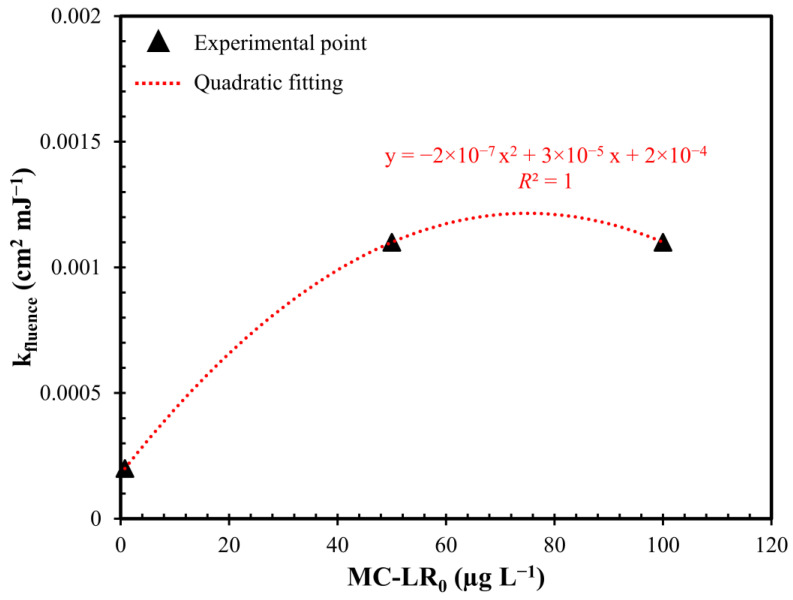
First-order kinetic constant (k_fluence_) as a function of the initial MC-LR concentration (MC-LR_0_). Conditions: H_2_O_2_ dosage = 0.3 mM; pH = 7.5 and fluence rate = 0.2 mW cm^−2^.

Comparing the apparent rate constant of the process as a function of the initial toxin concentration (Figure 5), the experimental points were well fitted by a second degree polynomial function that predicted an increase of MC-LR degradation kinetics when the initial concentration moved from 0 to 80 µg L^−1^. On the contrary, as the initial MC-LR increased after 80 µg L^−1^, a lowering of the kinetics of removal was detected. This result was confirmed in the literature by several studies where the lower kinetics, obtained with high MC-LR concentration, are linked to the increase of the internal optical density, which decrease the fraction of light absorbed by H_2_O_2_ limiting OH^•^ production [38,40,42]. On the contrary, with initial MC-LR concentration lower than 80 µg L^−1^, the obscuration of UV rays can be considered absent.

### 2.4. Energy Consumption

**Figure A3:**
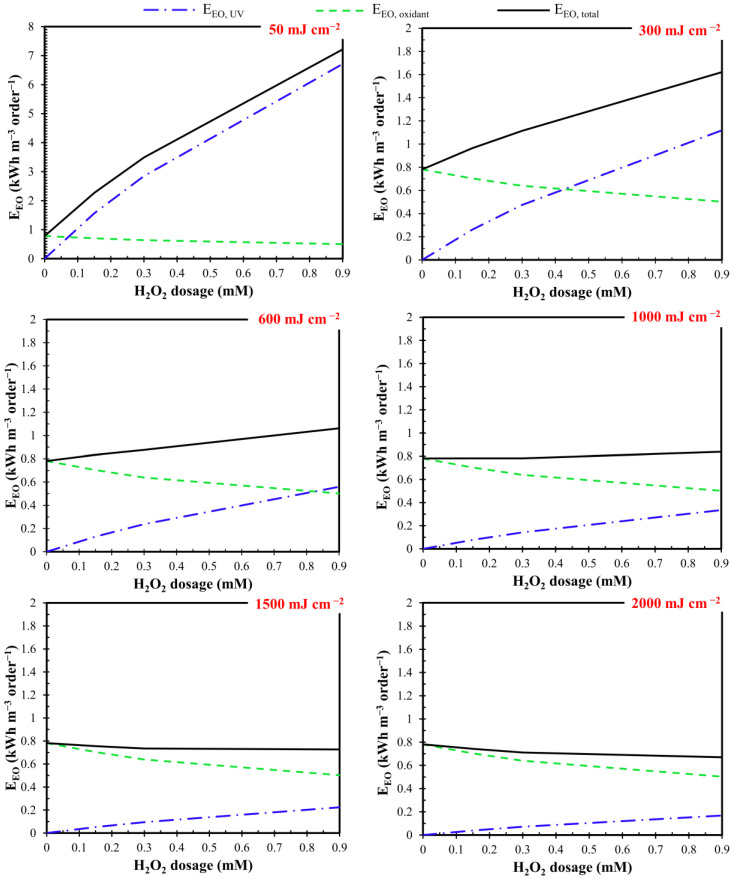
Electrical energy per order (E_EO_) as function of H_2_O_2_ dosage with different UV fluence. Conditions: MC-LR_0_ = 50 µg L^−1^; pH = 7.5 and fluence rate = 0.2 mW cm^−2^.

The specific energy consumption required to remove MC-LR by one order of magnitude (E_EO_) was assessed considering both the consumption given by the presence of UV (E_EO, UV_) and the use of H_2_O_2_ (E_EO, oxidant_). As shown in Figure A3 in Appendix A, electrical energy per order (E_EO_) was reported as a function of H_2_O_2_ dosage with tested UV fluence.

**Figure A4:**
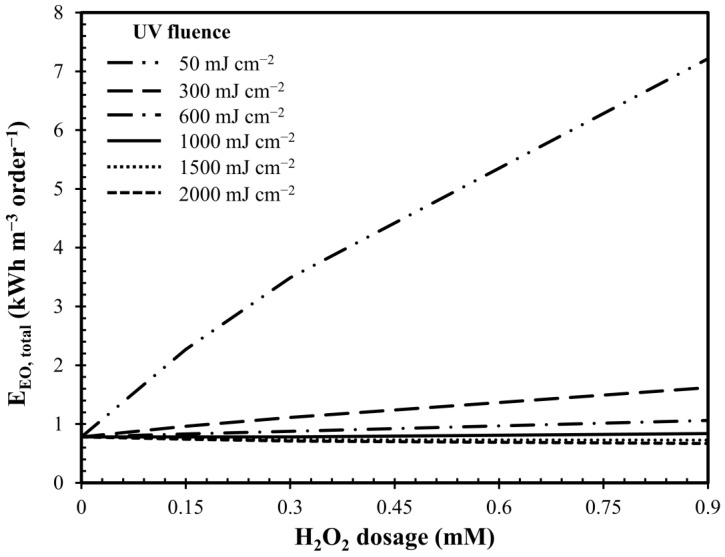
Total electrical energy per order (E_EO_), by different UV fluences, as a function of H_2_O_2_ dosage.

The results show that the total specific energy consumption (E_EO, total_) followed two different behaviors depending on the UV fluence considered and the H_2_O_2_ dosed (Figure A4 in the Appendix A). As the dosage of H_2_O_2_ increased, E_EO, total_ decreased with high UV fluences (1000 mJ cm^−2^, 1500 mJ cm^−2^ and 2000 mJ cm^−2^) while, in the presence of lower UV fluences (50 mJ cm^−2^, 300 mJ cm^−2^ and 600 mJ cm^−2^), E_EO, total_ increased even significantly as the concentration of the chemical oxidant dosed increased (Figure A4 in Appendix A).

In fact, significant H_2_O_2_ dosages in the presence of low UV radiation did not produce a significant increase in the effectiveness in removing MC-LR. On the contrary, by keeping the H_2_O_2_ dosage constant and increasing the fluence dose, the removal of MC-LR was more effective due to a greater production of OH^•^ radicals. Therefore, the specific energy consumption in relation to the MC-LR removed was lower.

**Figure 6:**
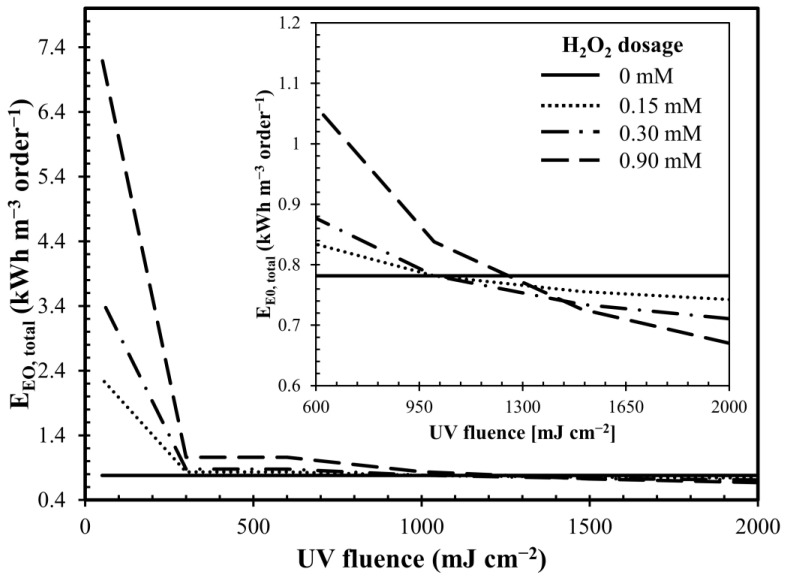
Total electrical energy per order (E_EO_), by different H_2_O_2_ dosage, as a function of UV fluence. Conditions: MC-LR_0_ = 50 µg L^−1^; pH = 7.5 and fluence rate = 0.2 mW cm^−2^.

By analyzing the behavior of E_EO, total_ as a function of UV fluence (Figure 6), the optimal dosage of H_2_O_2_ that minimized the total specific energy consumption related with MC-LR removed was identified. Operating at low fluence rates (lower than 950 mJ cm^−2^), UV alone allowed it to minimize the E_EO, total_. Operating between 950 and 1400 mJ cm^−2^, UV/H_2_O_2_ (0.3 µg L^−1^) combination minimized specific consumption while, for UV fluence higher than 1400 mJ cm^−2^, the combination UV/H_2_O_2_ (0.9 µg L^−1^) was the most advantageous due to higher MC-LR removal.

However, E_EO_ values strongly depend on the removal yields of the toxin and therefore on the production of OH^•^ radicals. In addition to the concentration of oxidants used, the production of hydroxyl radicals also depends on many other aspects including the hydrodynamics of the reactor and its configuration [46]. Therefore, a direct comparison with other research is difficult to make. However, although there are not many literature data on E_EO_ related to the removal of MC-LR and most of those reported do not evaluate the energetic consumption related to the use of oxidants, the values obtained are of the same order of magnitude as those reported by Schneider and Bláha (2020) [47].

## 3. Conclusions

The kinetics of the UV/H_2_O_2_ process was compared with those of UV and H_2_O_2_ showing the following result: UV/H_2_O_2_ > UV > H_2_O_2_. The UV/H_2_O_2_ combination allowed the removal of up to 93% of MC-LR (MC-LR_0_: 50 µg L^−1^; H_2_O_2_: 0.9 mM; UV fluence: 2000 mJ cm^−2^ and fluence rate: 0.2 mW cm^−2^). Within the range of H_2_O_2_ concentrations tested (0–0.9 mM), the results showed that H_2_O_2_ concentration and the removal kinetics followed a quadratic relation. By increasing the initial concentration of H_2_O_2_, the consumption of oxidant also increased but, in terms of MC-LR degraded for H_2_O_2_ dosed, the removal efficiency decreased. The initial concentration of MC-LR can significantly influence the kinetics of removal. The results showed that as the MC-LR_0_ increased, the removal kinetics increased, up to a limit concentration (80 µg L^−1^) in which the presence of high amounts of the toxin slowed down the process. About the specific energy consumption, UV alone minimized the specific energy consumption required when operating with UV fluence lower than 950 mJ cm^−2^. Operating between 950 and 1400 mJ cm^−2^, UV/H_2_O_2_ (0.3 mM) was the most advantageous combination while for UV fluence higher than 1400 mJ cm^−2^, the use of UV/H_2_O_2_ (0.9 mM) was the solution that involved lower energy consumption in relation to the quantity of MC-LR removed.

## 4. Materials and Methods

### 4.1. Water Preparation

In this study, powdered MC-LR (type ALX–350–012–C500; purity ≥ 95%; Enzo Life Sciences Farmingdale, NY, USA) was stored at −20 °C and used to prepare a 50 mg L^−1^ solution by adding 10 mL ethanol (≥99.8%) to 0.5 mg powdered MC-LR.

In order to better simulate conditions of treatment of a real DW, raw water was collected from Iseo Lake, in Peschiera Maraglio of Monteisola, in Northern Italy (province of Brescia, Lombardy) at 40 m depth and 40 m from the shore. Characteristics of raw water are reported in Table A2 in Appendix A. To separate dissolved MC-LR from cells, lake water samples were filtered using a 0.45 μm (pore size) glass fiber filter [48,49] and the permeate (MC-LR = 0.1 µg L^−1^) was spiked with the MC-LR solution to obtain toxin concentrations of 0.8 µg L^−1^, 50 µg L^−1^ and 100 µg L^−1^. Spiked waters were stored at 5 °C.

### 4.2. The Lab-Scale System

**Figure 7:**
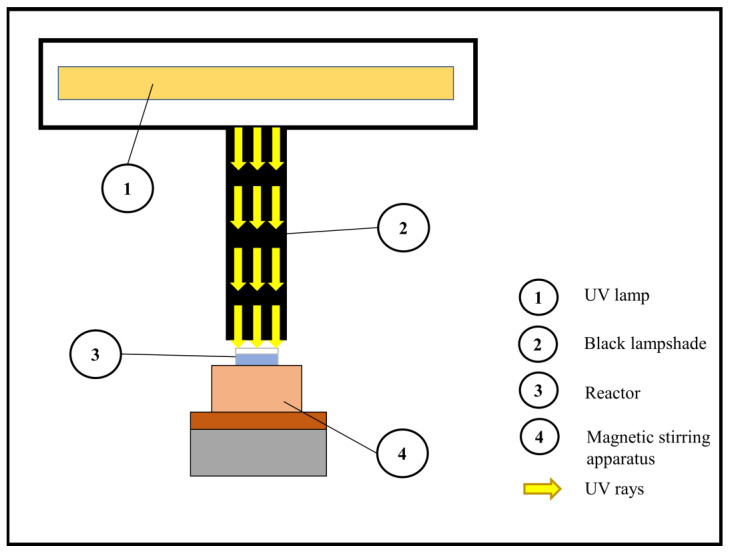
Scheme of the reactor used in test with UV, H_2_O_2_ and UV/H_2_O_2_.

The batch system used for experimental tests was composed as described in Figure 7. A low-pressure mercury UV lamp, which emits at 254 nm of wavelength, was used. A black lampshade avoided the dispersion of the light beams to the sides and allowed it to concentrate the radiation on the reactor. The intensity of the irradiation given by the UV-C rays incident on the reactor was 0.2 mW cm^−2^. A 50 mL Petri dish (diameter 5.45 cm, height 3.525 cm and thickness 0.25 cm), without lid, was used as a reactor. Inside the reactor, the water (depth 2.60 cm) was kept in constant stirring due to a magnetic stirring apparatus.

### 4.3. Experimental Set-Up and Analytical Methods

This study was carried out testing the following processes:

- H_2_O_2_ alone;
- UV alone;
- UV/H_2_O_2_ combination.

Tests were conducted at room temperature (22 ± 2 °C) and aimed to study the kinetics of MC-LR removal and investigate the effects of H_2_O_2_ dosage and initial MC-LR concentration.

Hydrogen peroxide (30%, *w*/*v*) was purchased from Carlo Erba Reagents S.r.l (Cornaredo, Lombardy, Italy) and, during tests, the residual concentration was measured using the triiodide method [50].

Before each experiment, to ensure a stable radiation, the UV lamps were allowed to warm up for 15 min. Fluence rate was measured with iodide/iodate actinometry method [51] and was equal to 0.2 mW cm^−2^.

pH value was monitored by means a portable multiparameter instrument (WTW 3410 SET4, Xylem Analytics Germany Sales GmbH, Weilheim, Germany).

After each fluence interval, samples were collected and catalase from *Micrococcus lysodeikticus* solution (Sigma Aldrich, St. Louis, MO, USA) was used to quench H_2_O_2_ reaction in samples before analysis according to Liu et al. [52]. The residual MC-LR concentration was measured with enzyme-linked immunosorbent assay (ELISA) kit, purchased from Eurofins Abraxis (Warminster, PA, USA). LOD and LOQ were equal to 0.1 µg L^−1^ and 5.0 µg L^−1^, respectively. The treated samples were diluted with Milli-Q water in order to obtain measurable values.

### 4.4. MC-LR Degradation

The results were elaborated according to a pseudo-first order kinetic as reported in Equation (1) [38]:

C = C_0_ × e^−kfluence^^× F^ (1)

where C_0_ represents the initial concentration of MC-LR and C is the current i-th value. k_fluence_ represents the apparent rate constant of the process (cm^2^ mJ^−1^) and F is the UV fluence (mJ cm^−2^). k_time_ (min^−1^) was calculated considering the fluence rate of the system (0.2 mW cm^−2^), and consequently half-life time (HLT) of MC-LR during treatments was found using the following equation [14]:

HLT = ln(2) × k_time_^−1^ (2)

### 4.5. Hydrogen Peroxide Consumption

Considering the amount of H_2_O_2_ consumed and the MC-LR removed, the H_2_O_2_ efficiency (mg mmol^−1^) was calculated according to Equation (3) [45]:

H_2_O_2_ efficiency = MC-LR_removed_ × H_2_O_2consumed_^−1^ (3)

### 4.6. Energy Consumption

When the concentration of the contaminant is very low, the amount of electric energy required to reduce the contaminant concentration by one order of magnitude can be considered independent of the initial concentration [46,53]. The water depth and the distance between the lamp and the water surface could affect the order of magnitude of the removal [46]. Although the lamp was not submerged into the reactor, in this work these two effects were neglected considering: (i) the low level of water inside the reactor and (ii) the presence of the black lampshade that avoided the dispersion of UV rays conveying them onto the reactor. Therefore, the energy consumption of the UV system was evaluated following the kinetic model of the electrical energy per order (E_EO_) according to Equation (4) [46,54,55]:

E_EO,UV_ = (P × t × 10^3^) × (V × log_10_(C_0_ C^−1^))^−1^ (4)

where P is the nominal power (kW) of the system, t (h) is the processing time and V (L) is the volume of water. The nominal power (P) was assumed equal to the energy input to the system, considering a fluence rate of 0.2 mW cm^−2^ and assuming a UV-C production yield of the lamp equal to 35%.

In view of the application on a larger scale, it is important to know not only the energy consumption necessary to produce UV-C but also the energy consumption due to the dosage of the oxidizing reagent (H_2_O_2_). In this work also, the chemical energy consumption associated to H_2_O_2_ was evaluated according to Equation (5) [56]:

E_EO,oxidant_ = (C_H__2O__2_ × CF) × (log_10_(C_0_ C^−1^))^−1^ (5)

where C_H__2O__2_ is the concentration of H_2_O_2_ (g m^−3^) and CF is a conversion factor equal to 6.67 × 10^−3^ kWh g^−1^ [56,57,58]. Therefore, total energy consumption can be calculated as reported in Equation (6):

E_EO,total_ = E_EO,UV_ + E_EO,oxidant_ (6)

## Abbreviations

AOPs	Advanced oxidation processes
DW	Drinking water
DWTP	Drinking water treatment plant
HLT	Half-life time
IARC	International Agency for Research on Cancer
LOD	Limit of detection
LOQ	Limit of quantification
MC-LR	Microcystin-LR
UV	Ultraviolet
WHO	World Health Organization

## Appendix A

**Table A1:** Kinetic constants and half-life times (HLT) of MC-LR during degradation by UV and UV/H_2_O_2_ using a first-order kinetics model. Conditions: MC-LR_0_ = 50 µg L^−1^; pH = 7.5; fluence rate = 0.2 mW cm^−2^ and *N* = number of total data.

	UV	UV/H_2_O_2_ (0.15 mM)	UV/H_2_O_2_ (0.30 mM)	UV/H_2_O_2_ (0.90 mM)
**R^2^ (-)**	0.977	0.974	0.985	0.963
**k_fluence_ (cm^2^ mJ)**	0.0009	0.001	0.0011	0.0014
**k_time_ (min^−1^)**	0.0108	0.0120	0.0132	0.0168
**HLT (min)**	64.2	57.8	52.5	41.3
***n* (-)**	12	7	16	7

**Table A2:** Kinetic constants and half-life times (HLT) of MC-LR during degradation by UV/H_2_O_2_, with different initial MC-LR concentration, using a first-order kinetics model. Conditions: H_2_O_2_ dosage = 0.3 mM; pH = 7.5; fluence rate = 0.2 mW cm^−2^ and *N* = number of total data.

	UV/H_2_O_2_ (0.8 µg L^−1^)	UV/H_2_O_2_ (50 µg L^−1^)	UV/H_2_O_2_ (100 µg L^−1^)
**R^2^ (-)**	0.940	0.990	0.956
**k_fluence_ (cm^2^ mJ)**	0.0002	0.0011	0.0011
**k_time_ (min^−1^)**	0.0024	0.0132	0.0132
**HLT (min)**	288.8	52.5	52.5
***n* (-)**	3	16	5

**Table A3:** Characteristics of the raw water before filtration.

Parameter	Unit of Measure	Average Value
pH	-	7.5
Dissolved oxygen	mg L^−1^	9.5–9.7
Turbidity	NTU	2–3
Absorbance UV at 254 nm	1 cm^−1^	0.010–0.020
Suspended solids	mg L^−1^	0–1
Conductivity at 20 °C	µS cm^−1^	260–270
Alkalinity	mg HCO_3_^−^ L^−1^	120–130
Bacteria colony count at 22 °C	CFU mL^−1^	80–90
Total coliforms at 37 °C	MPN 100 mL^−1^	65–75
*Enterococcus*	MPN 100 mL^−1^	2–4
*Escherichia coli*	MPN 100 mL^−1^	8–10
*Pseudomonas aeruginosa*	CFU 250 mL^−1^	2–4
*Clostridium perfringens*	CFU 100 mL^−1^	1–3
Cyanobacterial algae	cells L^−1^	1,700,000–2,000,000
Total algae	cells L^−1^	3,500,000–3,600,000

## References

[1] Sorlini S., Collivignarelli M.C., Carnevale Miino M. (2019). Technologies for the control of emerging contaminants in drinking water treatment plants. Environ. Eng. Manag. J..

[2] WHO (2017). WHO Guidelines for Drinking-Water Quality: Fourth Edition Incorporating the First Addendum.

[3] Leblanc P., Merkley N., Thomas K., Lewis N.I., Békri K., Renaud S.L., Pick F.R., McCarron P., Miles C.O., Quilliam M.A. (2020). Isolation and Characterization of [D-Leu1]microcystin-LY from Microcystis aeruginosa CPCC-464. Toxins.

[4] Sorlini S., Collivignarelli M.C., Abba A. (2018). Control Measures for Cyanobacteria and Cyanotoxins in Drinking Water. Environ. Eng. Manag. J..

[5] Kim S., KimiD S., Mehrotra R., Sharma A. (2020). Predicting cyanobacteria occurrence using climatological and environmental controls. Water Res..

[6] IARC (2010). IARC Monographs on the Evaluation of Carcinogenic Risks to Humans|Ingested Nitrate and Nitrite, and Cyanobacterial Peptide Toxins.

[7] Greer B., Meneely J.P., Elliott C.T. (2018). Uptake and accumulation of Microcystin-LR based on exposure through drinking water: An animal model assessing the human health risk. Sci. Rep..

[8] Yang S., Chen L., Wen C., Zhang X., Feng X., Yang F. (2018). MicroRNA expression profiling involved in MC-LR-induced hepatotoxicity using high-throughput sequencing analysis. J. Toxicol. Environ. Heal. Part A.

[9] Sun Y.T., Zheng Q., Huang P., Guo Z., Xu L.H. (2013). Microcystin-lr induces protein phosphatase 2a alteration in a human liver cell line. Environ. Toxicol..

[10] Zhou L., Yu H., Chen K. (2002). Relationship between microcystin in drinking water and colorectal cancer. Biomed. Environ. Sci..

[11] Wen C., Zheng S., Yang Y., Li X., Chen J., Wang X., Feng X., Yang F. (2019). Effects of microcystins-LR on genotoxic responses in human intestinal epithelial cells (NCM460). J. Toxicol. Environ. Heal. Part A.

[12] Yang F., Cao L., Massey I.Y., Yang F. (2020). The lethal effects and determinants of microcystin-LR on heart: A mini review. Toxin Rev..

[13] Xiang L., Li Y.W., Wang Z.R., Liu B.L., Zhao H.M., Li H., Cai Q.Y., Mo C.H., Li Q. (2020). Bioaccumulation and Phytotoxicity and Human Health Risk from Microcystin-LR under Various Treatments: A Pot Study. Toxins.

[14] Araújo M.K.C., Chia M.A., Arruda-Neto J.D.D.T., Tornisielo V.L., Vilca F.Z., Bittencourt-Oliveira M.D.C. (2016). Microcystin-LR bioaccumulation and depuration kinetics in lettuce and arugula: Human health risk assessment. Sci. Total Environ..

[15] EC Proposal for a Directive of the European Parliament and of the Council on the Quality of Water Intended for Human Consumption (Recast). https://eur-lex.europa.eu/resource.html?uri=cellar:8c5065b2-074f-11e8-b8f5-01aa75ed71a1.0016.02/DOC_2&format=PDF.

[16] IMH Interministerial Decree (2012). Scheme for the introduction. Annex I, Part B, of the Legislative Decree 2 February 2001, n. 31, of the “Microcystin-LR” Parameter and its Parameter Value (in Italian).

[17] Arnich N., Chorus I. (2012). FRANCE: Regulation, Risk Management, Risk Assessment and Research on Cyanobacteria and Cyanotoxins. Current Approaches to Cyanotoxin Risk Assessment, Risk Management and Regulations in Different Countries.

[18] Government of France (2007). Order of 11 January 2007 Relating to the Quality Limits and References for Raw Water and Water Intended for Human Consumption Mentioned in Articles R. 1321-2, R. 1321-3, R. 1321-7 and R. 1321-38 of Public Health Code.

[19] Government of Canada (2020). Guidelines for Canadian Drinking Water Quality; Water and Air Quality Bureau, Healthy Environments and Consumer Safety Branch, Health Canada.

[20] Government of Canada (2020). Guidelines for Canadian Recreational Water Quality—Cyanobacteria and their Toxins.

[21] Australian Government (2011). Australian Drinking Water Guidelines.

[22] Luo Z., Li P., Cai D., Chen Q., Qin P., Tan T., Cao H. (2017). Comparison of performances of corn fiber plastic composites made from different parts of corn stalk. Ind. Crop. Prod..

[23] Czyżewska W., Piontek M. (2019). The Efficiency of Microstrainers Filtration in the Process of Removing Phytoplankton with Special Consideration of Cyanobacteria. Toxins.

[24] Lürling M., Kang L., Mucci M., Van Oosterhout F., Noyma N.P., Miranda M., Huszar V.L., Waajen G., Marinho M.M. (2020). Coagulation and precipitation of cyanobacterial blooms. Ecol. Eng..

[25] Lama S., Muylaert K., Karki T.B., Foubert I., Henderson R.K., Vandamme D. (2016). Flocculation properties of several microalgae and a cyanobacterium species during ferric chloride, chitosan and alkaline flocculation. Bioresour. Technol..

[26] Jeong B., Oh M.S., Park H.M., Park C., Kim E.J., Hong S.W. (2017). Elimination of microcystin-LR and residual Mn species using permanganate and powdered activated carbon: Oxidation products and pathways. Water Res..

[27] Drogui P., Daghrir R., Simard M.-C., Sauvageau C., Blais J.F. (2011). Removal of microcystin-LR from spiked water using either activated carbon or anthracite as filter material. Environ. Technol..

[28] Mashile P.P., Mpupa A., Nomngongo P.N. (2018). Adsorptive removal of microcystin-LR from surface and wastewater using tyre-based powdered activated carbon: Kinetics and isotherms. Toxicon.

[29] Villars K., Huang Y., Lenhart J.J. (2020). Removal of the Cyanotoxin Microcystin-LR from Drinking Water Using Granular Activated Carbon. Environ. Eng. Sci..

[30] Moon B.R., Kim T.K., Kim M.K., Choi J., Zoh K.D. (2017). Degradation mechanisms of Microcystin-LR during UV-B photolysis and UV/H_2_O_2_ processes: Byproducts and pathways. Chemosphere.

[31] Park J.A., Yang B., Jang M., Kim J.H., Kim S.B., Park H.D., Park H.M., Lee S.H., Choi J.W. (2019). Oxidation and molecular properties of microcystin-LR, microcystin-RR and anatoxin-a using UV-light-emitting diodes at 255 nm in combination with H_2_O_2_. Chem. Eng. J..

[32] Park J.A., Yang B., Kim J.H., Choi J.W., Park H.D., Lee S.H. (2018). Removal of microcystin-LR using UV-assisted advanced oxidation processes and optimization of photo-Fenton-like process for treating Nak-Dong River water, South Korea. Chem. Eng. J..

[33] Zhu G., Lu X., Yang Z. (2015). Characteristics of UV-MicroO 3 Reactor and Its Application to Microcystins Degradation during Surface Water Treatment. J. Chem..

[34] Sorlini S., Biasibetti M., Collivignarelli M.C., Crotti B.M. (2015). Reducing the chlorine dioxide demand in final disinfection of drinking water treatment plants using activated carbon. Environ. Technol..

[35] Sorlini S., Collivignarelli M.C., Canato M. (2015). Effectiveness in chlorite removal by two activated carbons under different working conditions: A laboratory study. J. Water Supply Res. Technol..

[36] Sorlini S., Biasibetti M., Gialdini F., Collivignarelli M.C. (2016). How can drinking water treatments influence chlorine dioxide consumption and by-product formation in final disinfection?. Water Sci. Technol. Water Supply.

[37] Li W.Y., Liu Y., Sun X.L., Wang F., Qian L., Xu C., Zhang J.P. (2015). Photocatalytic degradation of MC-LR in water by the UV/TiO2/H2O2 process. Water Supply.

[38] He X., Pelaez M., Westrick J.A., O’Shea K.E., Hiskia A., Triantis T., Kaloudis T., Stefan M.I., De La Cruz A.A., Dionysiou D.D. (2012). Efficient removal of microcystin-LR by UV-C/H2O2 in synthetic and natural water samples. Water Res..

[39] Wang X., Utsumi M., Yang Y., Li D., Zhao Y., Zhang Z., Feng C., Sugiura N., Cheng J.J. (2015). Degradation of microcystin-LR by highly efficient AgBr/Ag3PO4/TiO2 heterojunction photocatalyst under simulated solar light irradiation. Appl. Surf. Sci..

[40] Liu J., Ye J.S., Ou H., Lin J. (2017). Effectiveness and intermediates of microcystin-LR degradation by UV/H2O2 via 265 nm ultraviolet light-emitting diodes. Environ. Sci. Pollut. Res..

[41] Collivignarelli M.C., Pedrazzani R., Sorlini S., Abbà A., Bertanza G. (2017). H2O2 Based Oxidation Processes for the Treatment of Real High Strength Aqueous Wastes. Sustainability.

[42] Li L., Gao N.Y., Deng Y., Yao J.J., Zhang K.J., Li H.J., Yin D.D., Ou H.S., Guo J.W. (2009). Experimental and model comparisons of H2O2 assisted UV photodegradation of Microcystin-LR in simulated drinking water. J. Zhejiang Univ. A.

[43] Vlad S., Anderson W.B., Peldszus S., Huck P.M. (2014). Removal of the cyanotoxin anatoxin-a by drinking water treatment processes: A review. J. Water Heal..

[44] Loaiza-González J.M., Salazar M.C.L., Rubio-Clemente A., Rodriguez D.C., Peñuela G., Salazar C.L., Rodríguez D.C., Peñuela G.A. (2019). Efficiency of the removal of microcystin-LR by UV-radiation and hydrogen peroxide. Revista Facultad de Ingeniería Universidad de Antioquia.

[45] Penru Y., Guastalli A.R., Esplugas S., Baig S. (2012). Application of UV and UV/H2O2 to seawater: Disinfection and natural organic matter removal. J. Photochem. Photobiol. A Chem..

[46] Keen O., Bolton J., Litter M., Bircher K., Oppenländer T. (2018). Standard reporting of Electrical Energy per Order (EEO) for UV/H2O2 reactors (IUPAC Technical Report). Pure Appl. Chem..

[47] Schneider M., Bláha L. (2020). Advanced oxidation processes for the removal of cyanobacterial toxins from drinking water. Environ. Sci. Eur..

[48] Grützmacher G., Böttcher G., Chorus I., Bartel H. (2002). Removal of microcystins by slow sand filtration. Environ. Toxicol..

[49] Jeon Y., Li L., Calvillo J., Ryu H., Domingo J.W.S., Choi O., Brown J., Seo Y. (2020). Impact of algal organic matter on the performance, cyanotoxin removal, and biofilms of biologically-active filtration systems. Water Res..

[50] Klassen N.V., Marchington D., McGowan H.C. (1994). H2O2 Determination by the I3- Method and by KMnO4 Titration. Anal. Chem..

[51] Rahn R.O., Bolton J., Stefan M.I. (2006). The Iodide/Iodate Actinometer in UV Disinfection: Determination of the Fluence Rate Distribution in UV Reactors. Photochem. Photobiol..

[52] Liu W., Andrews S., Stefan M.I., Bolton J.R. (2003). Optimal methods for quenching H2O2 residuals prior to UFC testing. Water Res..

[53] Collivignarelli M.C., Abbà A., Miino M.C., Arab H., Bestetti M., Franz S. (2020). Decolorization and biodegradability of a real pharmaceutical wastewater treated by H_2_O_2_-assisted photoelectrocatalysis on TiO_2_ meshes. J. Hazard. Mater..

[54] Malpass G.R.P., Miwa D., Mortari D., Machado S., Motheo A. (2007). Decolorisation of real textile waste using electrochemical techniques: Effect of the chloride concentration. Water Res..

[55] Farkas J., Náfrádi M., Hlogyik T., Pravda B.C., Gajda-Schrantz K., Hernádi K., Alapi T. (2018). Comparison of advanced oxidation processes in the decomposition of diuron and monuron–efficiency, intermediates, electrical energy per order and the effect of various matrices. Environ. Sci. Water Res. Technol..

[56] Zhang R., Yang Y., Huang C.-H., Zhao L., Sun P. (2016). Kinetics and modeling of sulfonamide antibiotic degradation in wastewater and human urine by UV/H_2_O_2_ and UV/PDS. Water Res..

[57] Yao H., Sun P., Minakata D., Crittenden J.C., Huang C.-H. (2013). Kinetics and Modeling of Degradation of Ionophore Antibiotics by UV and UV/H_2_O_2_. Environ. Sci. Technol..

[58] Sun P., Tyree C., Huang C.-H. (2016). Inactivation of Escherichia coli, Bacteriophage MS2, and Bacillus Spores under UV/H_2_O_2_ and UV/Peroxydisulfate Advanced Disinfection Conditions. Environ. Sci. Technol..

